# Skin health in oncology: evidence-based skin care for cancer patients

**DOI:** 10.1093/oncolo/oyag194

**Published:** 2026-06-01

**Authors:** Alexander Kaminsky, Sara Suhl, Yoni Sacknovitz, Gary K Schwartz, Sandhya Deverapalli, Larisa J Geskin

**Affiliations:** Columbia University Vagelos College of Physicians and Surgeons, New York, NY 10032, United States; Columbia University Vagelos College of Physicians and Surgeons, New York, NY 10032, United States; Columbia University Vagelos College of Physicians and Surgeons, New York, NY 10032, United States; Case Comprehensive Cancer Center, Cleveland, OH 44106, United States; Tufts Medical Center, Boston, MA 02111, United States; Department of Dermatology, Columbia University Irving Medical Center, New York, NY 10032, United States

**Keywords:** oncology, skin care, sun protection, moisturization, review

## Abstract

**Background:**

Cancer and its treatments frequently compromise skin integrity, leading to xerosis, pruritus, dermatitis, radiation-induced skin damage, and secondary infections. These cutaneous adverse events (cAEs) are not merely superficial; they profoundly impact patient quality of life (QoL), contributing to physical discomfort, psychosocial distress, and poorer treatment adherence. Even mild skin toxicities elevate dermatology-specific QoL measures, affecting mood and social function. Additionally, new targeted therapies, such as daraxonrasib—a KRAS inhibitor under development for pancreatic cancer—can cause a dose-limiting acneiform facial rash.

**Methods:**

In this study, relevant literature on skin care in cancer patients was identified using PubMed. Evidence from clinical studies, reviews, and expert guidelines was summarized.

**Results:**

Evidence supports proactive skincare with gentle pH-balanced cleansers, ceramide- or urea-based moisturizers, and broad-spectrum photoprotection to lessen symptom severity and improve QoL. Sun protection remains essential because chemotherapy and immunomodulatory therapies increase photosensitivity, accelerate photoaging, and raise the risk of secondary skin cancers. Supportive skincare interventions have also shown emotional and functional benefits, including improved mood and self-image during chemotherapy and radiation treatment.

**Conclusion:**

Despite strong data supporting moisturization, barrier repair, and photoprotection, oncologic treatment plans often overlook these interventions. By integrating dermatologic education and skincare maintenance into standard oncologic practice, we can minimize cAEs, foster treatment compliance, and enhance patients’ physical comfort and psychosocial well-being. This narrative review synthesizes mechanistic insights, clinical evidence, and practical recommendations for oncologists to champion comprehensive skincare in all cancer patients, whether directly affected by skin issues or not.

Implications for Practice This manuscript highlights how fundamental skincare steps—such as moisturizing and sun protection—can make a tremendous difference for cancer patients. Treatments and diseases often cause painful or visible skin problems that hurt quality of life and may disrupt care. By including safe, evidence-based skincare in cancer treatment plans, doctors can help patients feel more comfortable, stay on therapy longer, and maintain a stronger sense of self during a difficult time.

## Introduction

The skin, the body’s largest organ, is a dynamic and essential interface between internal physiology and the external environment. In oncology, the skin functions as both a target and a barometer of disease, sustaining direct involvement from malignancy while also reflecting the effects of systemic therapies. Yet despite its critical role in physical protection, thermoregulation, immune surveillance, and psychosocial identity, skin care remains an under-addressed component of comprehensive cancer care.

Cutaneous adverse events (cAEs) are common in cancer patients. The incidence of dermatologic toxicity in patients undergoing chemotherapy or targeted therapies has been reported as high as 45%-85%; these toxicities range from xerosis, pruritus, and photosensitivity to severe erosive dermatitis, hand-foot syndrome, or bullous eruptions.^1–4^ These manifestations are not merely cosmetic; they disrupt sleep, provoke intense discomfort, reduce social functioning, and can delay or limit essential oncologic therapy.^5–7^ Moreover, patients whose skin is not visibly affected by their disease or treatment may suffer from impaired barrier function, heightened sensitivity, or accelerated photoaging, making proactive skincare a universal need.

Incorporating structured skin care, particularly moisturization and sun protection, has been shown to improve symptoms, reduce treatment interruptions, and enhance quality of life. Scientific evidence highlights the importance of maintaining acidic skin pH (4.5-5.5), repairing ceramide-based lipid barriers, preventing transepidermal water loss (TEWL), and supporting a stable skin microbiome.^8–12^ Meanwhile, photosensitivity induced by oncologic agents increases the risk for secondary skin cancers and mandates vigilant photoprotection.^13–15^

Despite these findings, oncologists rarely receive formal education on skin health, and skincare counseling is inconsistently applied in cancer care. This narrative review provides a clinically applicable framework for integrating evidence-based skin care into oncologic practice, with a focus on patient comfort, adherence, and long-term skin preservation.

## Skin biology in health and disease

**Figure 1.**
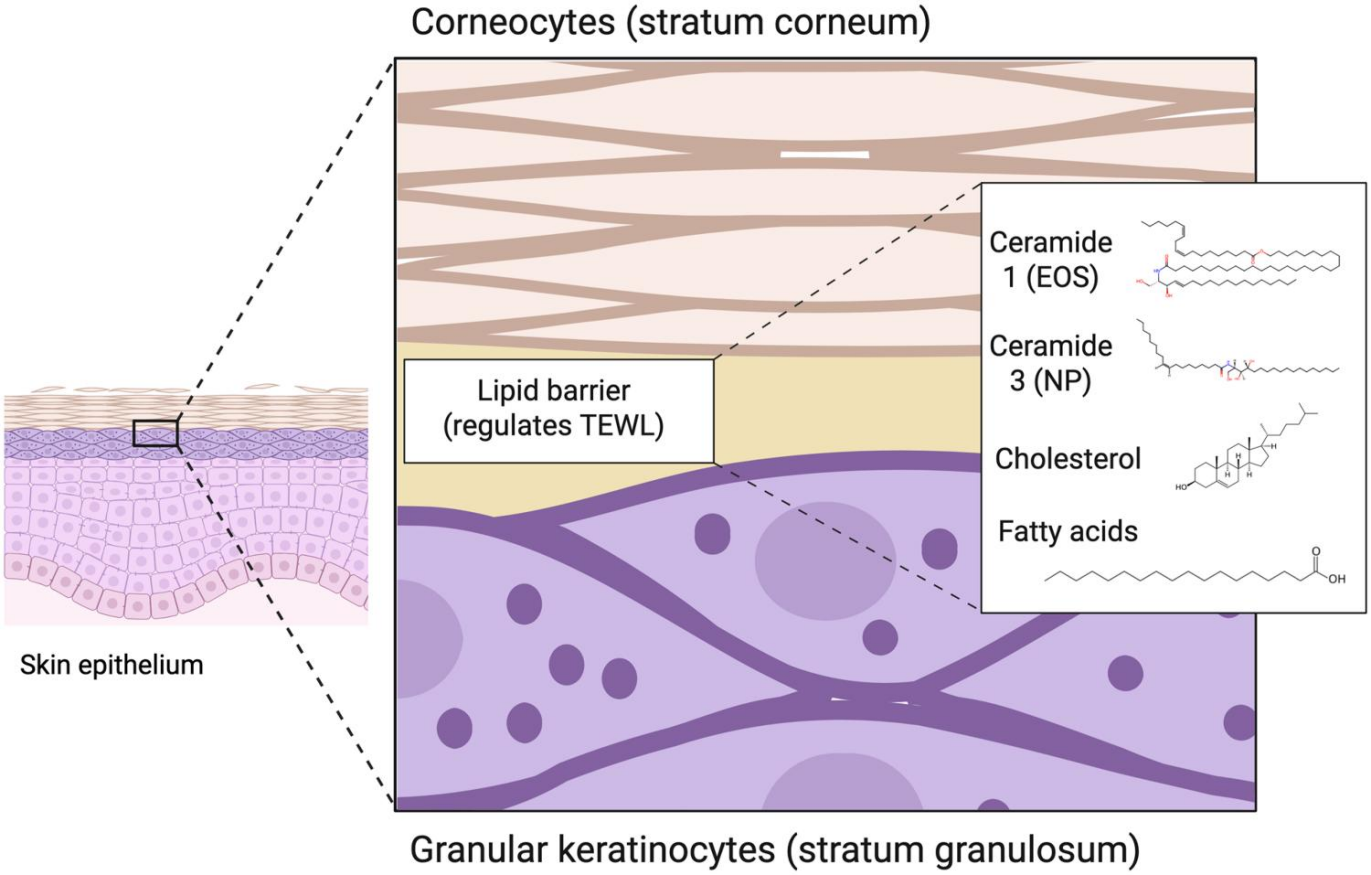
Lipids in the stratum corneum.

The skin is a vital, multifunctional organ that protects the body from mechanical injury, microbial invasion, dehydration, and ultraviolet radiation. Structurally, its outermost layer, the stratum corneum, functions as a biologic barrier composed of corneocytes embedded in a highly ordered lipid matrix rich in ceramides, cholesterol, and free fatty acids (see Figure 1). This matrix regulates TEWL and provides resilience against external insults. Underlying keratinocytes, immune cells, vasculature, and nerve endings contribute to thermoregulation, immune surveillance, and sensory perception.^8^^,^^16^

A hallmark of healthy skin is its acidic surface pH, which supports lipid processing, maintains protein function, and activates antimicrobial peptides. This acid mantle also helps shape the composition of the cutaneous microbiome, which prevents pathogen colonization and modulates inflammation.^9^^,^^11^^,^^17^ Any disruption from aging, illness, or treatment can lead to impaired barrier function and higher infection risk.

In oncology patients, the skin is often affected by therapies targeting rapidly dividing cells or immune pathways. Chemotherapy agents (such as taxanes, anthracyclines, and 5-fluorouracil) impair epidermal turnover, damage the lipid matrix, and suppress sebaceous gland activity. Reductions in ceramide synthesis and alterations in skin pH contribute to a cascade of barrier dysfunction and inflammation, manifesting as xerosis, fissuring, and heightened photosensitivity.^1^^,^^4^^,^^18^

Several chemotherapeutic agents (including pegylated liposomal doxorubicin, capecitabine, and 5-fluorouracil) have also been implicated in a distinct constellation of clinical and histopathological findings affecting the acral surfaces known as hand-foot syndrome (HFS).^19^ Patients report palmoplantar dysesthesia followed by tenderness, erythema, and edema, often within days or weeks of initiating therapy.^19^

**Figure 2.**
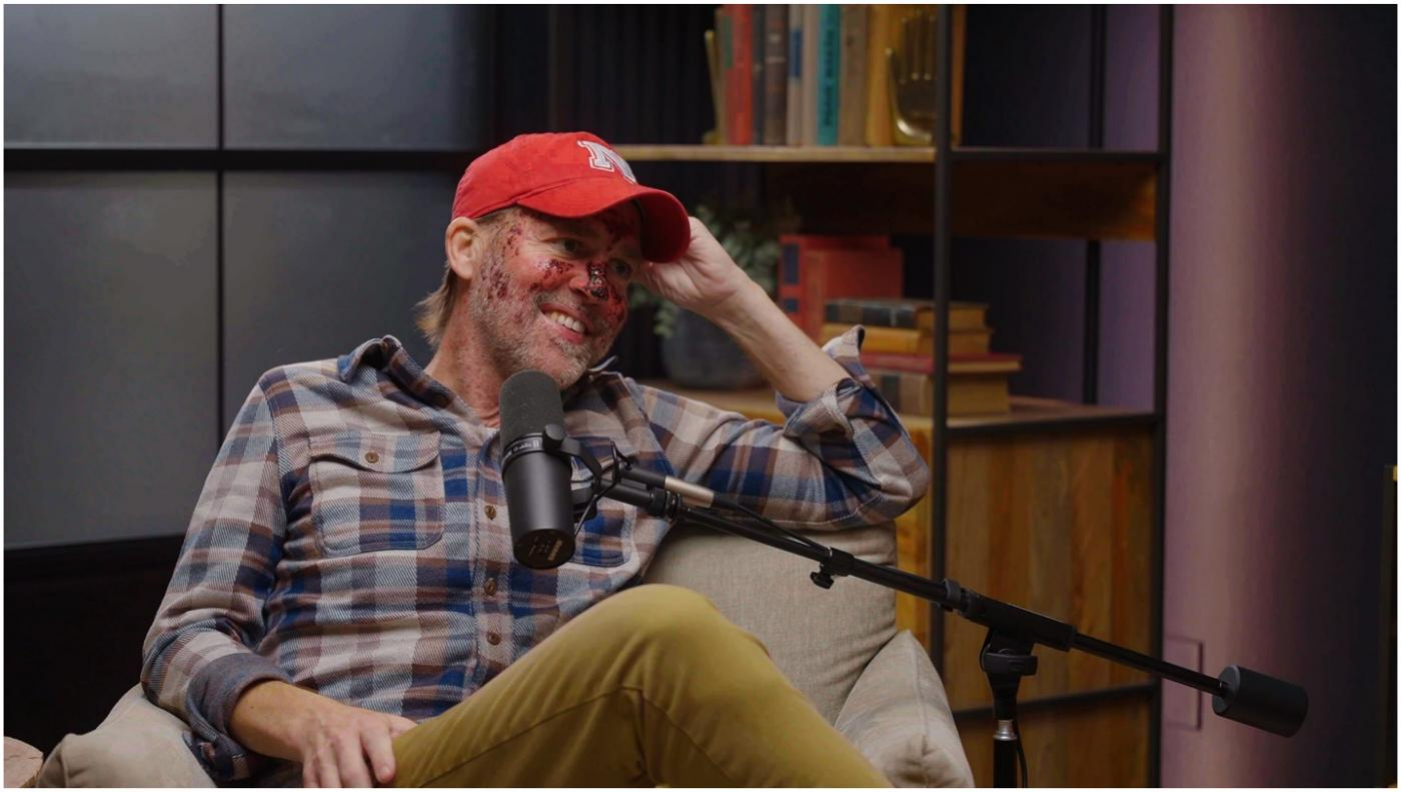
Severe cutaneous reaction to daraxonrasib therapy in a patient with pancreatic cancer (Photo credit: The New York Times/Redux,^64^ reprinted with permission from the New York Times).

Targeted therapies, such as EGFR inhibitors, are well known to induce an acneiform face rash that can significantly affect patients’ quality of life and treatment adherence. A similar rash has been seen in patients receiving experimental KRAS inhibitors^20^; in a recent article in the New York Times, former United States senator Ben Sasse recently described living with the cutaneous effects of daraxonrasib (Figure 2, NY Times).

**Figure 3.**
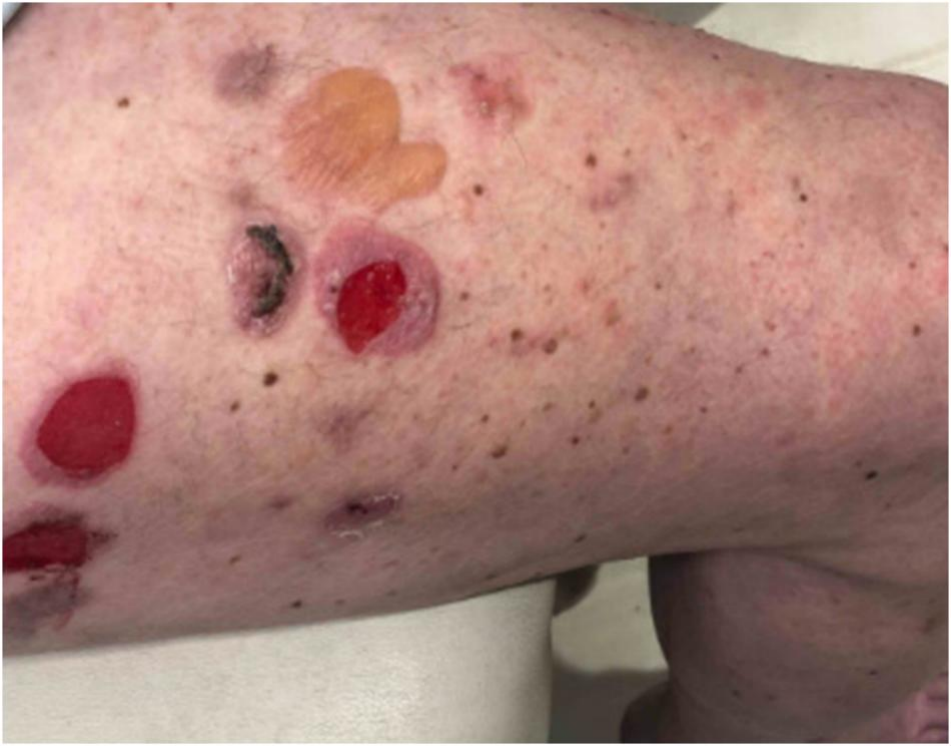
Lichen planus pemphigoides/bullous pemphigoides secondary to pembrolizumab.

Another significant drug class is immunotherapy targeting the PD-1/PD-L1 and CTLA-4 pathways, which have revolutionized cancer treatment but often result in immune-related cAEs. These include maculopapular rashes, vitiligo-like depigmentation, lichenoid reactions, and bullous pemphigoid (Figure 3). These eruptions reflect inappropriate activation of T cells against skin antigens and often correlate with systemic immune toxicities.^21–23^ Importantly, such dermatologic reactions may serve as early indicators of broader immune dysregulation. As discussed further below, referral to dermatology is recommended in all cases of severe skin reactions.

Radiation therapy similarly disrupts skin integrity in a dose- and time-dependent fashion. Acute radiation dermatitis is characterized by erythema, edema, and desquamation due to basal keratinocyte death, vascular compromise, and oxidative stress. Chronic effects, including fibrosis, atrophy, and pigmentary changes, result from impaired stem cell renewal and vascular remodeling.^2^^,^^24^ These changes compromise local immune surveillance and wound healing, particularly in previously irradiated skin or in areas with compromised vascular supply.

In parallel, emerging evidence underscores the role of skin microbiome in regulating immune responses and maintaining barrier integrity; cancer therapies can perturb this microbiome, often favoring colonization with pathogenic species. For example, in CTCL, there is evidence that *S. aureus* produces superantigens that drive T-cell activation and disease flares. In immunosuppressed patients, microbial shifts may result in candidiasis, cellulitis, and delayed wound healing.^25^^,^^26^

A summary of commonly reported cAEs, grouped according to therapy type, is shown in Table 1. Altogether, these therapeutic disruptions affect cellular regeneration, lipid synthesis, pH homeostasis, immune regulation, and microbial balance and create a skin environment prone to inflammation, infection, and breakdown. Recognizing and managing these changes proactively is essential for symptom control, treatment adherence, and prevention of complications.

**Table 1:** Common cutaneous adverse events secondary to antineoplastic therapy.

Therapy type	Common examples	Common cAEs and basic management approach
**Platinum compounds**	Cisplatin, carboplatin	Photosensitivity: sunscreen (SPF 50+, UVA/UVB), photoprotective clothing, avoid peak sun.
**Antimetabolites**	5-Fluorouracil, capecitabine	Xerosis: twice-daily moisturizer, gentle skin care (see Figure 5). Pruritus: moisturization, oral antihistamines. Photosensitivity: sunscreen (SPF 50+, UVA/UVB), photoprotective clothing, avoid peak sun. HFS: moisturization, topical steroids, analgesics, avoid heat and friction, gentle skin care (see Figure 5).
**Protein kinase inhibitors**	Vemurafenib, vandetanib	Photosensitivity: sunscreen (SPF 50+, UVA/UVB), photoprotective clothing, avoid peak sun. Xerosis: twice-daily moisturizer, gentle skin care (see Figure 5). Paronychia: nail care, topical steroids.
**EGFR inhibitors**	Osimertinib, cetuximab	See Table 2 for management algorithm. Papulopustular (acneiform) rash: prophylaxis with oral tetracyclines, moisturizers, sun protection; treatment with topical steroids, topical antibiotics (systemic if severe). Xerosis: twice-daily moisturizer, gentle skin care (see Figure 5). Paronychia: nail care, topical steroids.
**KRAS inhibitors**	Sotorasib, adagrasib In development: daraxonrasib (RMC-6236, see Figure 2), RMC-6291, zoldonrasib	See Table 2 for management algorithm. Photosensitive hyperpigmentation: sunscreen (SPF 50+, UVA/UVB), photoprotective clothing, avoid peak sun. Papulopustular (acneiform) rash: prophylaxis with oral tetracyclines, topical steroids; treatment with topical steroids, topical antibiotics (systemic if severe).
**Immune checkpoint inhibitors**	PD-1/PD-L1 inhibitors	Photosensitivity reactions and pigment changes: sunscreen (SPF 50+, UVA/UVB), photoprotective clothing, avoid peak sun. Lichenoid eruptions (Figure 3): topical steroids, oral antihistamines; may require course of systemic corticosteroids or drug discontinuation if severe.
**Anthracyclines**	Doxorubicin, daunorubicin, epirubicin	Xerosis: twice-daily moisturizer, gentle skin care (see Figure 5). Photosensitivity: sunscreen (SPF 50+, UVA/UVB), photoprotective clothing, avoid peak sun. HFS (pegylated liposomal doxorubicin): moisturization, topical steroids, analgesics, avoid heat and friction, gentle skin care (see Figure 5). Alopecia: gentle hair and skin care (see Figure 5), cooling caps.
**Taxanes**	Paclitaxel, docetaxel	Xerosis: twice-daily moisturizer, gentle skin care (see Figure 5). Photosensitivity: sunscreen (SPF 50+, UVA/UVB), photoprotective clothing, avoid peak sun. Alopecia: gentle hair and skin care (see Figure 5), cooling caps.
**Radiation therapy**		Erythema: moisturization, topical steroids. Desquamation: non-adhesive dressings, wound care. Chronic changes can include fibrosis, skin atrophy, pigmentary changes. Ulceration/necrosis: dermatology or wound care referral.

## Impact on treatment and quality of life

Cancer therapies often precipitate cAEs that compromise patients’ physical, emotional, and social functioning. Unlike systemic toxicities measured by laboratory or imaging findings, skin toxicity is visible, affecting daily comfort, self-perception, and interpersonal interactions. Studies using validated patient-reported outcome measures such as the Dermatology Life Quality Index (DLQI) and Skindex-16/17 consistently show that skin involvement—including xerosis, pruritus, HFS, and dermatitis—significantly lowers QoL scores, even when adverse events are clinically graded as low severity.

Such symptoms can limit a patient’s ability or willingness to continue therapy; indeed, observational studies have demonstrated that unmanaged or inadequately treated cAEs are a significant non-oncologic reason for treatment interruption.^27^^,^^28^ In some cases, diagnostic uncertainty or concern for serious cutaneous reactions leads to precautionary treatment holds or permanent discontinuation, potentially compromising treatment intensity and oncologic outcomes.

**Figure 4.**
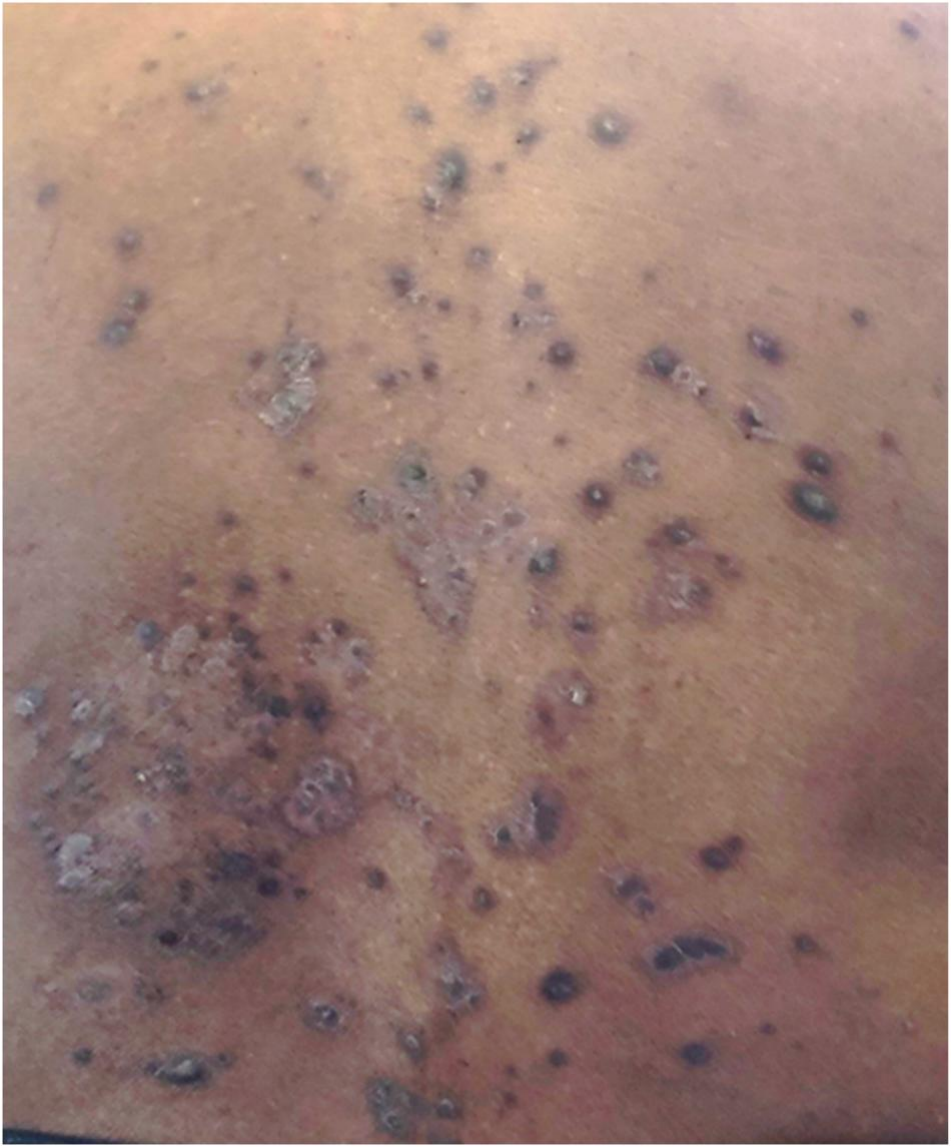
Psoriasis secondary to pembrolizumab.

In a retrospective analysis involving 73 patients referred for dermatologic evaluation following cancer treatment interruption, over 80% responded to dermatologic treatment and subsequently resumed therapy.^29^ Similarly, a 2020 retrospective study of 47 patients with suspected immune checkpoint inhibitor (ICI)-related cAEs found that dermatologic consultation significantly reduced both ICI discontinuation and systemic corticosteroid use.^30^ This is particularly relevant in patients receiving ICIs, which can induce de novo inflammatory and autoimmune dermatologic conditions—including lichen planus pemphigoides/bullous pemphigoides (Figure 3) and psoriasis (Figure 4)—these are often diagnostically and therapeutically challenging without specialized dermatologic input. These findings highlight the role of specialized skin care in preserving treatment continuity, as reflected in expert consensus.^31^

Supportive skin care interventions have also demonstrated measurable QoL benefits. In a randomized controlled trial involving breast cancer patients undergoing radiotherapy, prophylactic application of a heparinoid moisturizer led to significant reductions in the worsening of the DLQI “Symptoms & Feelings” subscale over a 4-week period, as compared to no intervention.^32^ Although mean total DLQI scores remained comparable between arms, the moisturizer group exhibited faster resolution of symptoms post-treatment, with improved subjective comfort and functional recovery.

A prospective study of 99 patients receiving chemotherapy or radiotherapy provided participants with a standardized skincare regimen consisting of a tailored moisturizer, facial cleanser, and face cream and demonstrated a significant reduction in Skindex-16 scores by week 4.^33^ Improvements were noted in domains such as dryness, discomfort, and appearance-related distress. Importantly, the intervention was well-tolerated and universally accepted, emphasizing the practical feasibility of such regimens in routine oncology care.

Beyond moisturization, several studies have highlighted the psychosocial benefits of structured cosmetic and holistic skincare programs. In a randomized, controlled trial, breast cancer patients undergoing chemotherapy and radiation received adjunctive aesthetic care consisting of manicure/pedicure, gentle massage, and dermocosmetic emollient therapy. They reported significantly improved mood state and self-perception of the disease by the end of the treatment period.^34^ Another study of women with cancer therapy-related skin changes found that usage of camouflage makeup resulted in improved cancer-specific QoL metrics regardless of age, diagnosis, and site of skin changes.^35^ These benefits extended beyond the dermatologic, reinforcing skin care’s role in restoring autonomy and dignity during an otherwise disempowering experience.

Mechanistically, these improvements are not simply psychological in origin. Skincare interventions are biologically grounded: emollients restore depleted lipids, reduce TEWL, maintain an acidic skin pH (critical for enzymatic and antimicrobial function), and reduce the permeability of irritants.^8^ The resulting decrease in subclinical inflammation correlates with reduced sensory discomfort and visual skin changes—both strongly associated with patient distress and QoL impairment.^36^

Given the wealth of research on the subject, current guidelines advocate for routine skincare during cancer therapy. Recommendations include proactive daily moisturization, use of pH-balanced cleansers, avoidance of irritants (fragrances, alcohols, and parabens), and early dermatologic consultation at the first signs of toxicity.^37^^,^^38^ Despite this, in our experience, implementation in oncology practice remains uneven, often relying on patient-initiated efforts or ad hoc nurse guidance.

Taken together, the existing evidence establishes a strong scientific and clinical rationale for incorporating skin health management into the standard of care in oncology. Simple, accessible interventions such as gentle cleansing, regular moisturization, and cosmetic support improve both physical symptoms and psychosocial outcomes. In doing so, they affirm a model of cancer care that values the patient’s total experience and not just disease metrics.

## Mechanistic rationale for moisturization

As mentioned above, the integrity of the stratum corneum is fundamental to maintaining skin health. Disruption of this barrier can lead to xerosis, fissuring, pruritus, and increased susceptibility to infection and inflammation.^16^ Effective moisturization—targeting hydration, lipid replenishment, and TEWL prevention—involves humectants, emollients, and/or occlusives. Humectants such as urea, glycerin, and hyaluronic acid attract water from the dermis and external environment into the stratum corneum. Urea, at concentrations greater than 5%, not only hydrates but also exhibits mild keratolytic properties that smooth the skin surface and reduce scaling.^39^ In oncology patients, where barrier lipid synthesis is often suppressed by cytotoxic therapies, humectants are vital to re-establishing stratum corneum hydration.^8^

Emollients, including squalene, plant-derived oils, and isopropyl myristate, fill the microscopic gaps between desquamating corneocytes. This reduces friction, restores pliability, and improves surface texture. Importantly, certain botanical emollients possess anti-inflammatory properties, contributing to overall barrier resilience.^40^ Their role is particularly pronounced in patients receiving epidermal growth factor receptor (EGFR) inhibitors, where skin fragility and erythema are common.^41^

Occlusive agents are “sealants,” which form a barrier over the skin and include petrolatum, dimethicone, and lanolin derivatives; these compounds form a semi-impermeable layer that reduces TEWL by as much as 98% after application.^42^ This function is critical in irradiated or chemotherapeutically damaged skin, where the stratum corneum is often markedly disrupted.^43^ Occlusives not only prevent water evaporation but also allow underlying humectants and emollients to act more effectively by maintaining a hydrated microenvironment.

Ceramides restore lipid lamellae, an endogenous source of moisturization. Cancer therapies, particularly alkylating agents and radiation, deplete EOS (ceramide 1) and NP (ceramide 3) and undermine the lipid bilayers that form the mortar of the stratum corneum.^44^ Ceramide-rich moisturizers have been shown to rapidly restore lamellar structure and normalize barrier function. In murine and human studies, topical ceramides improved hydration, reduced inflammatory cytokine expression, and enhanced epidermal turnover rates in damaged skin.^45^

Clinical studies have validated these mechanisms. In randomized trials, moisturizers containing combinations of humectants, ceramides, and occlusives have normalized hydration metrics within days, reduced TEWL, and significantly improved patient-reported comfort.^46^ These findings justify moisturization not merely as symptomatic relief, but as a scientifically grounded therapeutic strategy integral to supportive cancer care.

## Sun protection in oncology

Photosensitivity is a common and underappreciated complication of many cancer therapies, including chemotherapy, targeted agents, immunotherapies, and radiation. In the context of compromised skin barrier function and immunomodulatory drugs, UV exposure during treatment not only heightens the risk of acute phototoxicity such as sunburn and dermatitis but also amplifies long-term risks of photoaging, mutagenesis, and secondary skin malignancies (see below).

UV radiation can be subdivided into several wavelength ranges. UVB is directly absorbed by DNA and interferes with replication.^47^ UVA penetrates deeper into the dermis and generates reactive oxygen species that induce indirect DNA damage, lipid peroxidation, and mitochondrial dysfunction.^48^ These mutagenic events are compounded in patients receiving cisplatin, fluorouracil, or vemurafenib, which sensitize skin to UV-induced cytotoxicity.^49^ Resident Langerhans cells are particularly sensitive to UVB and undergo depletion upon exposure, impairing local immune surveillance and reducing DNA repair capacity.^50^

A growing number of oncologic agents are now recognized as photosensitizers, either via direct phototoxicity or immune dysregulation (see Table 1). For oncology patients, physical sunblocks containing zinc oxide and titanium dioxide are strongly preferred. These inorganic compounds reflect both UVA and UVB rays and exhibit minimal allergenicity or systemic absorption.^51^ In contrast, chemicals such as oxybenzone, avobenzone, and octinoxate absorb UV radiation and dissipate it as heat. Although effective, these compounds may pose risks of contact dermatitis, endocrine disruption, and environmental toxicity.^52^ Oxybenzone, in particular, has demonstrated systemic absorption and has been detected in urine and plasma after a single application, though clinical implications remain under debate.^53^ Regardless of the sunscreen type, addressing both UVA and UVB radiation is essential, as both can contribute to the development of skin cancers and photosensitivity.^54^

Sunscreens should offer broad-spectrum coverage (UVA/UVB), high SPF (>50) for immunocompromised users, water resistance (ideally for 40-80 minutes), and minimal fragrance and alcohol content to prevent irritant reactions. Current guidelines recommend applying at least 2 mg/cm^2^ of sunscreen to exposed areas 15 minutes before exposure and reapplying every 2 hours, particularly after sweating or swimming.^55^

Additionally, several emerging agents and formulations hold promise for enhanced photoprotection in oncology. Polypodium leucotomos extract, a natural fern-derived antioxidant, reduces UV-mediated DNA damage and improves skin phototype tolerance in patients with photosensitive conditions.^56^ DNA repair enzymes embedded in liposomes are being tested as post-exposure topical agents.^57^ Finally, smart textiles and UPF-rated clothing, particularly those incorporating UV-reflective fibers, offer an important non-topical layer of defense for photosensitive patients.^58^

Additionally, oncologic photoprotection should include avoidance of sun exposure between 10 AM and 4 PM; seeking shade; wearing appropriate sun-protective clothing; and patient education on UV-reflective surfaces such as water, sand, and snow.

There is evidence that structured sun safety education improves adherence to therapy and influences attitudes toward UV exposure.^59^ For oncology patients, sun protection is more than a cosmetic concern: it is a medical imperative. The interplay of UV radiation with photosensitizing drugs, immunosuppression, and impaired skin barrier function necessitates a rigorous, evidence-based approach. A combination of physical sunscreens, protective clothing, and patient education represents best practice. As innovations in sunscreen formulation and photomedicine evolve, oncologists must remain vigilant and proactive in guiding their patients toward effective, safe, and individualized sun protection strategies.

## Clinical algorithm and recommendations for skin care in oncology

Cutaneous toxicities are among the most frequent adverse effects of systemic cancer therapies, yet structured skin care interventions remain inconsistently applied in oncologic practice. A proactive, evidence-based skin care algorithm can mitigate cAEs, improve patient comfort, prevent treatment delays, and support adherence to anticancer therapies. This section proposes a clinical algorithm for skin care in oncology, derived from dermatology and oncology literature and adaptable to general practice.

At treatment initiation, all oncology patients should undergo a baseline dermatologic risk assessment, including skin type (Fitzpatrick I–VI), history of atopy or prior radiation dermatitis, and comorbidities such as diabetes or autoimmune disorders. Medication review should focus on agents known to cause photosensitivity or xerosis. This step allows clinicians to identify vulnerable individuals and tailor preventative strategies accordingly.

Cleansing routines should preserve the acid mantle, essential for microbiome balance and enzymatic lipid processing. pH-balanced, fragrance-free cleansers are recommended, while alkaline soaps and harsh surfactants such as sodium lauryl sulfate should be avoided. Moisturization should begin before dryness becomes clinically evident; the recommended regimen includes twice-daily moisturizer application, preferably within 3 minutes of bathing.

As discussed above, photoprotection is essential for oncology patients, including those receiving regimens not traditionally associated with phototoxicity. This precaution applies even to newer targeted therapies such as KRAS inhibitors, a drug class first approved in 2021^60^; notably, a 2024 case report described sotorasib-associated photosensitive hyperpigmentation, underscoring that unexpected cutaneous photoreactive effects may still occur with these agents.^61^ Physical sunscreens containing zinc oxide or titanium dioxide provide broad-spectrum protection with minimal allergenicity. SPF > 50 is recommended, with application 15 minutes before sun exposure and reapplication every 2 hours. UPF-rated clothing and avoidance of peak sunlight hours further reduce UV exposure risks.

Prophylactic measures should also be considered for medications that have a known rash association. For example, administration of EGFR inhibitors, which can classically produce an acneiform (papulopustular) rash, is often paired with prophylactic moisturization, oral tetracyclines, and topical steroids.^62^ The same is true of the novel KRAS inhibitor class,^63^ particularly G12D and G12X inhibitors. A representative clinical photograph in Figure 2 demonstrates severe facial erosions that developed within several months of initiating therapy with daraxonrasib, a pan-KRAS inhibitor.^64^ A management guide for these medication classes has been provided in Table 2.

**Table 2:** Management recommendations for papulopustular and inflammatory cutaneous toxicities associated with EGFR-, KRAS-, and MAPK-pathway inhibitor therapies.

CTCAE grade	Clinical features	Management strategies	Escalation/additional considerations
**Prophylaxis**	Prior to initiation of EGFR-, KRAS-, or MAPK-pathway inhibitor therapy	Preventative skin care including gentle cleanser, thick moisturizer twice daily, broad-spectrum sunscreen, and avoidance of irritants/fragranced products (Figure 5). Consider prophylactic oral doxycycline 100 mg BID or oral minocycline 100 mg daily/BID during the first 4-6 weeks of therapy.	Educate patients to report rapidly progressive rash, pain, drainage, blistering, mucosal lesions, or fever promptly.
**Grade 1**	<10% BSA involvement, minimal symptoms	Continue anticancer therapy at full dose. Continue preventative skin care. Oral tetracycline-class antibiotic therapy. Low-potency topical corticosteroid to face/intertriginous areas. Medium-potency topical corticosteroid to trunk/extremities. Oral nonsedating antihistamines as needed.	Reassess within 1-2 weeks.
**Grade 2**	10%-30% BSA involvement; pruritus, tenderness, or symptoms limiting instrumental activities of daily living	Continue therapy in most cases; consider dose interruption for intolerable, progressive, or refractory symptoms. Continue Grade 1 measures. Consider topical clindamycin for papulopustular/acneiform eruption. Consider bacterial culture and treatment if crusting, drainage, or concern for secondary infection is present.	Close monitoring and dermatology referral for persistent, atypical, infected, or worsening eruption.
**Grade 3**	>30% BSA involvement; severe symptoms limiting self-care activities of daily living	Interrupt therapy. Resume treatment (often at reduced dose) after improvement to Grade ≤1. Medium- or high-potency topical corticosteroids. Consider short course of systemic corticosteroids for severe inflammatory or eczematous eruptions after exclusion of active infection. For recurrent or refractory cases, consider oral isotretinoin (papulopustular eruption) or dupilumab (eczematous phenotype).	Dermatology referral strongly recommended. Evaluate and treat secondary bacterial infection if present. May require permanent drug discontinuation if recurrent or severe.
**Grade 4/SCAR concern**	Life-threatening eruption, extensive desquamation, blistering, mucosal involvement, skin pain, necrosis, facial edema, purpura, or systemic symptoms (fever/eosinophilia)	Immediately discontinue therapy. Hospitalization and urgent dermatology evaluation. Evaluate for SJS/TEN, DRESS, AGEP, bullous pemphigoid, or severe infection. Initiate supportive care and phenotype-directed therapy.	Permanent discontinuation is generally required unless an alternative diagnosis is established.

Abbreviations: BID = twice daily; BSA = body surface area; CTCAE = Common Terminology Criteria for Adverse Events; SCAR = severe cutaneous adverse reaction.

For patients with skin erosions, ulcers, or treatment-induced wounds, wound care should include saline or vinegar soaks, followed by the application of hydrocolloid or hydrogel dressings. These create a moist environment conducive to re-epithelialization. Low-potency topical corticosteroids can be added in the presence of inflammation.

Management of dermatologic toxicity should follow a graded response approach. Mild symptoms may be managed with moisturizers and antipruritics. Moderate inflammation warrants topical corticosteroids, while severe reactions (such as bullae or vasculitis) require dermatologic consultation and potential therapy modification. The Common Terminology Criteria for Adverse Events, utilized by the NCI, can be referenced to help determine AE severity. In patients with severe skin toxicities, cessation of the culprit drug and systemic immunosuppression is generally recommended; for example, expert guidelines for severe ICI-induced bullous pemphigoid suggest several weeks of systemic steroids, followed by (if necessary) doxycycline and niacinamide, rituximab, mycophenolate mofetil, methotrexate, or azathioprine.^23^ For HFS (see above), mainstays of treatment include drug discontinuation or dose modification in combination with topical steroids and wound care; without these interventions, the condition can lead to keratoderma.^19^

**Figure 5.**
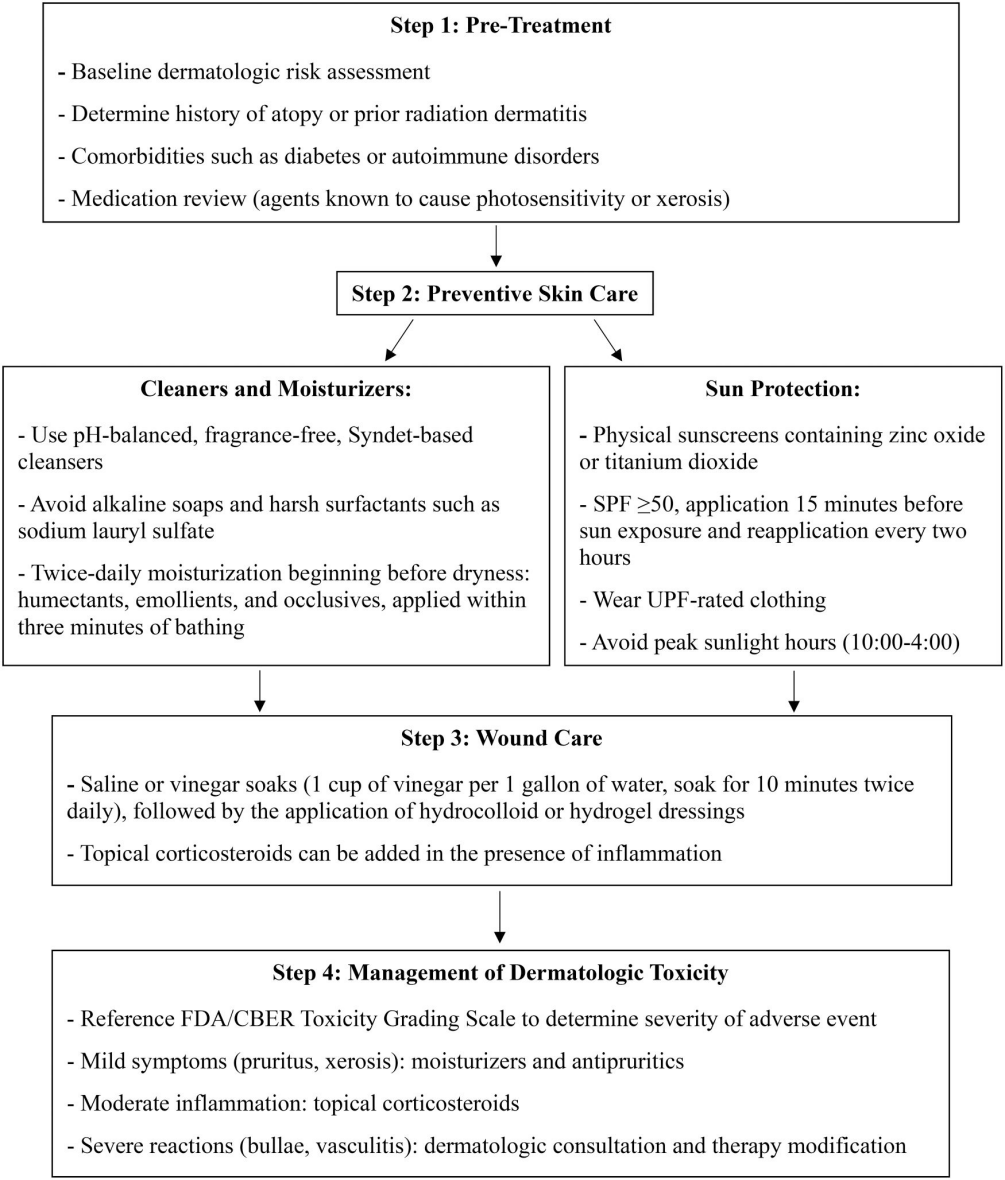
Stepwise recommendations for skin care in oncology.

Patient and staff education are central to long-term adherence. Instructional materials, skin care demonstrations, and regular follow-up reinforce best practices. Routine skin checks should be integrated into oncology visits, particularly for high-risk therapies such as EGFR or VEGF inhibitors and checkpoint blockade. An example stepwise general skin care algorithm is provided in Figure 5.

## Implementation in oncologic practice

The high prevalence and distressing nature of skin toxicities during cancer treatment make dermatologic support a clinical priority, not a luxury. However, implementation must be pragmatic and compatible with existing oncology workflows to succeed. Effective integration of skin care into oncologic practice requires a systems-based approach involving team education, patient counseling, and process embedding.

Oncology care teams should be equipped with foundational knowledge about dermatologic toxicities. EMR-integrated “smartphrases” or pocket guides tailored to common agents (such as EGFR inhibitors and ICIs)^65^^,^^66^ can assist with bedside implementation. Collaboration with dermatology should be formalized via co-management protocols and referral pathways, especially for high-risk patients or those with severe or refractory toxicity.^67^ Early referral to a dermatologist for evaluation of skin findings may reduce the risk of unnecessary interruptions in anti-cancer therapy, both in inpatient^68^ and outpatient settings^29^; if possible, such referrals should be directed to dermatologists that specialize in oncologic care. Pharmacy teams contribute essential insight into affordable ingredient-based options and methods to navigate insurance approval.^69^ Starter skin care kits, including cleansers, ceramide-based moisturizers, and physical sunscreen samples, can be distributed at treatment initiation; a prospective, descriptive study of 100 head and neck cancer patients given skin care kits and diaries revealed consistent treatment use.^70^

Skin care should be introduced as a proactive part of cancer therapy, similar to neutropenia precautions^31^ or nausea prevention. Visual demonstrations (eg, showing the patient the amount of cream used per body part) paired with written instructions improve understanding and compliance. Encourage ingredient recognition over brand loyalty: patients should learn to identify glycerin, ceramides, urea, petrolatum, and zinc oxide. Digital support tools such as Medisafe or CareZone help maintain medication routines through reminders.^71^

## Conclusion and call to action

Skin health is an essential, yet often overlooked, component of comprehensive cancer care. cAEs caused by chemotherapy, immunotherapy, radiation, and targeted agents significantly compromise patients’ comfort, dignity, and quality of life. These skin toxicities are not merely cosmetic; they are among the most visible and psychologically distressing side effects of treatment and can negatively impact adherence, cause treatment delays, and diminish patient trust in their care team.

Despite their prevalence, proactive strategies to prevent or manage skin side effects are rarely standardized in oncology workflows. This gap represents a critical opportunity for improvement. Evidence from clinical trials strongly supports that structured skin care centered around hydration, moisturization, and sun protection reduces symptom burden and enhances emotional well-being. When incorporated into care pathways from the outset, these interventions can transform how patients experience cancer therapy.

## Data Availability

All data discussed in this review are contained within the cited literature.
